# A Pilot Study of All-Computational Drug Design Protocol–From Structure Prediction to Interaction Analysis

**DOI:** 10.3389/fchem.2020.00081

**Published:** 2020-02-12

**Authors:** Yifei Wu, Lei Lou, Zhong-Ru Xie

**Affiliations:** Computational Drug Discovery Laboratory, School of Electrical and Computer Engineering, College of Engineering, University of Georgia, Athens, GA, United States

**Keywords:** ligand-protein docking, molecular dynamics simulation, computational drug discovery, SK2, structural prediction, binding site prediction, virtual mutation, pharmaceutical industry

## Abstract

Speeding up the drug discovery process is of great significance. To achieve that, high-efficiency methods should be exploited. The conventional wet-bench methods hardly meet the high-speed demand due to time-consuming experiments. Conversely, *in silico* approaches are much more efficient for drug discovery and design. However, *in silico* approaches usually serve as a supportive role in research processes. To fully exert the strength of computational methods, we propose a protocol which integrates various *in silico* approaches, from *de novo* protein structure prediction to ligand-protein interaction simulation. As a proof of concept, human SK2/calmodulin complex was used as a target for validation. First, we obtained a predicted structure of SK2/calmodulin and predicted binding sites which were consistent with the literature data. Then we investigated the ligand-protein interaction via virtual mutagenesis, flexible docking, and binding affinity calculation. As a result, the binding energies of mutants have similar trends compared with the EC_50_ values (*R* = 0.6 for NS309 in V481 mutants). The results indicate that our protocol can be applied to the drug design of structure unknown proteins. Our study also demonstrates that the integration of *in silico* approaches is feasible and it facilitates the acceleration of new drug discovery.

## 1. Introduction

To discover a new drug is an urgent but time-consuming process (Zhou et al., 2016; Gómez-Bombarelli et al., 2018). In the process of new drug development, *in silico* approaches have been successfully exploited to perform multiple simulations, such as selecting drug candidates from a database via high throughput virtual screening (Aparna et al., 2014; de Ruyck et al., 2016; Vilar et al., 2016; Imam and Gilani, 2017). The application of *in silico* approaches not only speeds up new drug discovery, but also collects related information, reveals the mechanisms and proposes new hypotheses. Compared to bench research, computational experiments perform high-efficiency simulations which considerably reduce the research time and the cost of experiments (Abel et al., 2017). However, in most cases, *in silico* approaches played an assisting role in the process. In this study, we propose an all-computational protocol integrating multiple *in silico* approaches to simulate the entire drug discovery process from *de novo* protein structure prediction to drug-protein interaction disclosure.

Up to now, reliable *in silico* approaches, such as molecular dynamics simulation (MD simulation) and machine learning (Durrant and McCammon, 2011; Lavecchia, 2015; Mortier et al., 2015), have been increasingly developed and applied in finding new drugs and optimizing them for treatment of diseases. However, most research projects only use one single *in silico* approach. For instance, the homology models of CIB2, a calcium- and integrin- binding protein, were constructed based on CIB1 structures using SWISS-MODEL server (Waterhouse et al., 2018). Based on the models, the way how the point mutations affect the affinities of calcium- and integrin-binding were predicted and then validated by *in vitro* experiments (Riazuddin et al., 2012). Besides, the results of ligand-protein docking were used to test the substrate specificity of OCT-1 and OCT-2 (organic cation transporter) to guide the following *in vivo* experimental validation (Papaluca and Ramotar, 2016). These works simply employed the *in silico* approaches as supportive methods, which did not sufficiently leverage the high-speed advantages of computational methods. Integrating the established *in silico* researches into an all-computation pipeline and producing validated good results is a milestone in the “omics” era.

Human small conductance calcium-activated (SK2) ion channels, consisted of SK2 subunits and calmodulin molecule, have been proved to be therapeutic targets for treatment of neuronal diseases, such as Parkinson's and amyotrophic lateral sclerosis (ALS) (Bond et al., 2005; Lu et al., 2009). The crystalized structures of human SK2 bound with Riluzole, an approved drug for ALS (Romano et al., 2015), and an analog [pdb (Berman et al., 2003) ligand ID: 658] of the SK2 activator CyPPA, an anti-ataxic agent (Herrik et al., 2012), have been released recently. It was also reported that ligands of two different chemical classes, Riluzole and its analog NS309, and CyPPA and its analogs, all bind to the same binding site where the interface of SK2 and calmodulin is. In addition, two key residues in the binding site were mutated to investigate how different mutations affect the potency of two ligands (Cho et al., 2018). As a proof of concept, we chose SK2 as the target to examine the protocol we proposed. Hence, in this study, we basically repeated the entire procedure of the previous study of Cho et al. (2018) with consecutive *in silico* approaches and compared our results with those of the bench experiments. We first constructed the human SK2/calmodulin model (PSK2) using SWISS-MODEL server and docked the ligands Riluzole and CyPPA analog 1 (PDB ligand ID: 658, see section 2.1), onto the predicted binding site. The predicted site is consistent with the reported results (Cho et al., 2018). Then the residues V481 and A477 in the binding pocket were virtually mutated and the mutation effects were assessed via binding energies (MM-GBSA ΔG_Bind_) calculation. The results show that the binding energies of mutants have similar trends compared with the EC_50_ (the concentration of a drug that gives half-maximal response) values (*R* = 0.6 for NS309 in V481 mutants). Overall, our results suggest that this protocol of *in silico* approach can provide a systematic prediction on the unknown structures of proteins and potential drugs, and they also demonstrate the ability of *in silico* approaches to speed up the new drug design process.

## 2. Materials and Methods

### 2.1. Protein Structure Preparation

Having a determined or predicted structure of the drug target protein is the first prerequisite of structure-based drug design. To prove the all-computational protocol is valid, we started our process from structure prediction. To predict the structure of SK2 and calmodulin from *Homo sapiens*, its amino acid sequence was obtained from the UniProt website (uniprot ID: Q9H2S1). Then we uploaded the sequence onto the SWISS-MODEL server, the most widely-used and reliable structure prediction server, to build a structure model (Bienert et al., 2016; Waterhouse et al., 2018) and selected the 3-D complex structure of *Rattus norvegicus* SK2 ion channels with NS309 (PDB ID: 4J9Z) (Zhang et al., 2013) as the template of structure prediction. 4J9Z was downloaded from RCSB's Protein Data Bank (Berman et al., 2003), and the predicted structure of SK2 and calmodulin were combined in Maestro (11.5 version, Schrodinger). To test the accuracy of modeling, we uploaded the predicted protein structure and the crystalized complex structure of human SK2 and calmodulin with Riluzole (PDB ID: 5V02) onto Zhang's web server (Zhang and Skolnick, 2005) to calculate the TM-align score. 5V02 was downloaded from RCSB's Protein Data Bank. In addition, site-directed mutants were constructed using the Mutate Residue function of Maestro.

A PDB structure of the target protein cannot be directly used in molecular docking without preprocessing. In most of the cases, a PDB file does not include the information of hydrogens, the (potential) charges of atoms, or the bond orders between any two atoms. In addition, the protein structures may be determined with a missing fragment(s), a low resolution or alternate positions, or under an unnatural condition, for example, low or high pH values. To make sure molecular docking can simulate the binding between ligands and the target protein correctly and precisely, the protein and ligand preparation is necessary. The wild-type and mutant (predicted) protein structures to be used for docking were processed by protein preparation wizard in Maestro (Sastry et al., 2013). The workflow of protein preparation contains three steps as follows: (1) Preprocess: assigning bond orders, adding hydrogens, creating zero-order bonds to metals, creating disulfide, filling in missing side chains using Prime, deleting waters beyond 5.00 Å from het groups (to keep the water molecules which may form hydrogen-bond bridges between the protein and the ligand and remove those cannot form hydrogen-bond bridges) and generating het states using Epik (pH = 7.0 ± 2.0) (Shelley et al., 2007); (2) Optimization: setting pH = 7.0 and performing optimization; (3) Minimization: this step was performed using the OPLS3 force field (Harder et al., 2015). The converge heavy atoms to root-mean-square deviation (RMSD) is 0.30 Å.

### 2.2. Ligand Preparation

The 3-D molecular structures of Riluzole and NS309 were obtained from the PubChem database (Kim et al., 2018). The 3-D molecular structures of CyPPA analog 1 ((4-chloro-phenyl)-[2-(3,5-dimethyl-pyrazol-1-yl)-pyrimidin-4-yl]-amine) and analog 2 (4-chloro-phenyl)-[2-(3,5-dimethyl-pyrazol-1-yl)-6-methyl-pyrimidin-4-yl]-amine) were built in Maestro (11.5 version, Schrodinger) based on a previous study (Cho et al., 2018). All the compounds were prepared using OPLS3 force field in Ligprep panel in Maestro (Sastry et al., 2013; Harder et al., 2015). The preparation process included converting 2D structures to 3D ones, adding hydrogens, computing correct partial charges, and optimizing the structures.

### 2.3. Binding Site Prediction

Knowing the potential ligand binding site(s) is also an important prerequisite prior to molecular docking. There are many well-developed binding site prediction methods and servers (Xie and Hwang, 2015); however, the predictions produced by different methods may disagree with each other. Therefore, researchers usually compare the prediction results produced by different methods to find the consensus among all predictions. In our study, binding site prediction process was completed using the LISE web server (http://lise.ibms.sinica.edu.tw/applet/) (Xie and Hwang, 2012), which is reported to have the highest accuracy (80–90% for a soluble protein), and the binding site prediction tool, Sitemap, in Maestro, which we used in the docking procedure after this step. The SK2 predicted structure was uploaded onto the LISE web server for binding site prediction. The top three predictions from LISE were then downloaded and imported into Maestro. Meanwhile, in Maestro, SiteMap (Halgren, 2007, 2009) predicted five ligand binding sites. After comparing the results obtained using two prediction methods, we used the consensus region to define the docking grid box.

### 2.4. Ligand-Protein Docking

In order to predict the details of the interaction between ligands and the target protein and to estimate their binding affinities (see section 2.5), ligand docking was conducted the extra-precision (XP) mode in Maestro. Maestro has three precision options for docking including high throughput virtual screening (HTVS), standard precision (SP), and extra precision (XP). Users can choose an option based on their need or the computational load. After the ligands and the target proteins were processed using Ligprep and protein preparation, respectively, a receptor grid box was generated according to the results of binding site prediction. The size of the receptor grid box was set as default. To investigate the interaction of the protein and ligands, Induced Fit Docking (IFD) (Farid et al., 2006; Sherman et al., 2006a,b; Clark et al., 2016) was performed in Maestro. Using the IFD, the Ligprep outputs were imported and docked to the target protein. The standard protocol was applied to generate up to 20 poses. The force field was OPLS3. Under the prime refinement tab, the conformations of binding site residues within 5 Å (default value) of the ligand were refined. In the Glide redocking process, the energy of each protein/ligand complex structure and the number of top structures were set as the default settings. The XP mode was used for all IFD process.

### 2.5. MM-GBSA Calculation

The binding energy (ΔG_Bind_) between a protein and a ligand reflects how stable they bind to each other and how a point mutation affects the ligand binding. Therefore, we examined if our model can correctly predict the trend of binding affinity changes of the mutations on the target protein. In this study, ΔG_Bind_ were estimated using the Prime MM-GBSA module in Maestro (Greenidge et al., 2012). In MM-GBSA panel, the pose viewer files of docked complex were uploaded. The solvation model was VSGB and the force field was OPLS3 (Li et al., 2011). Prime MM-GBSA ΔG_Bind_ was calculated using this equation:

(1) $$\begin{array}{l} \Delta {\text{G}}_{\text{Bind}}={\text{E}}_{\text{complex}}(\text{minimized})-[{\text{E}}_{\text{ligand}}(\text{minimized})\\ \;+\;{\text{E}}_{\text{receptor}}(\text{minimized})] \end{array}$$

Where ΔG_Bind_ is binding free energy and E_complex_(minimized), E_ligand_(minimized), and E_receptor_(minimized) are minimized energies of receptor-ligand complex, ligand and receptor, respectively.

### 2.6. Virtual Mutation

Based on the literature, valine 481 (V481) of SK2 is a crucial residue which forms the hydrophobic core between SK2 and calmodulin (Cho et al., 2018). To investigate the impacts of V481 mutations in the binding pocket using *in silico* approaches, we first implemented the site-directed mutagenesis in Maestro. The V481 was virtually mutated to alanine, serine, threonine, aspartate, or phenylalanine. Then NS309 and CyPPA analog 2 were docked onto the mutated binding site of PSK2 using IFD and the binding free energies were calculated using MM-GBSA to reveal the effects of mutated residues.

Alanine 477 (A477) is another vital residue in the binding pocket (Cho et al., 2018). We exploited the same method mentioned above to virtually mutate A477 to isoleucine, leucine, valine, serine, threonine, arginine, and aspartate, docked NS309 and CyPPA analog 2 onto the mutated binding site of PSK2 and calculated the binding free energies using MM-GBSA.

### 2.7. Molecular Dynamics Simulation

The molecular dynamics (MD) simulations were performed using GROMACS version 2018.1 and CHARMM36 all-atom force field (March 2019) (Vanommeslaeghe et al., 2010, 2012; Vanommeslaeghe and MacKerell, 2012; Yu et al., 2012; Gutiérrez et al., 2016). The starting coordinates of each protein-ligand complex were obtained from docking experiments. Then we defined a dodecahedral unit cell and filled it with water molecules. After adding ions, the complex was minimized for 50,000 steps of steepest descent minimization. Next, the complex was equilibrated using an NVT ensemble (constant Number of particles, Volume, and Temperature) and NPT ensemble (the Number of particles, Pressure, and Temperature). The target temperature for equilibration was 300 K. At last, the simulations were performed for 30 ns. After the MD simulations, we calculated the RMSD of the residues which were mentioned in the previous research in four trajectories (Cho et al., 2018). Then, we selected four time points of two residues: I100 on protein-NS309 complex (15,000, 18,000, 24,000, and 30,000 ps) and D64 on protein-CyPPA analog 1 (2,610, 6,000, 15,000, and 21,000 ps). Finally, four conformations of both residues were converted into PDB files and were superposed using PyMol.

## 3. Results

### 3.1. Structure Prediction of Human SK2/Calmodulin Complex

**Figure 1:**
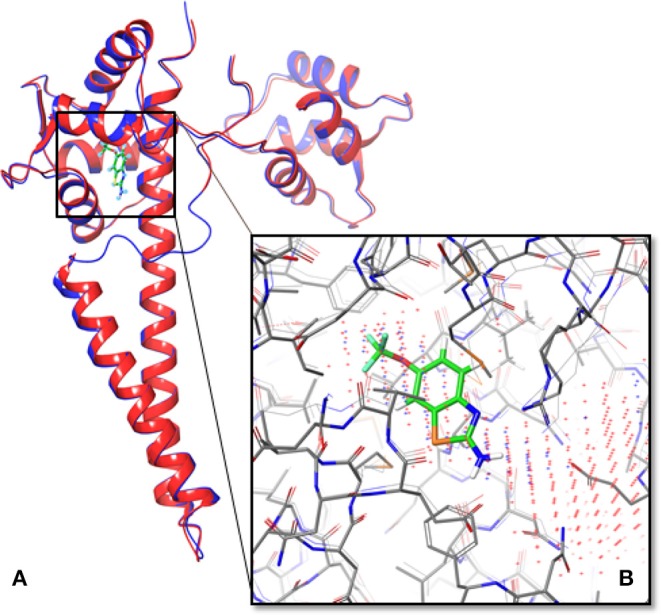
Overlapped structures of the predicted model of human SK2 ion channels and 5V02. **(A)** The predicted structure of human SK2 ion channels (PSK2) in blue overlapped with its crystallized structure (5V02) in red. The structure of a missing loop (IDF) near the ligand binding pocket in 5V02/5V03 has also been predicted. The green compound was Riluzole, which was the ligand in 5V02. **(B)** The predicted binding sites (Blue dots represent the results from Sitemap; red dots represent the results from LISE) overlapped with the Riluzole binding site in 5V02.

The structure of human SK2/calmodulin was generated using template-based modeling on the SWISS-MODEL server (Figure 1A). Based on the structure of SK2/calmodulin from *Rattus norvegicus* (PDB ID: 4J9Z), the structure models were predicted using the amino acid sequences of human SK2 and calmodulin. Additionally, the loop (residue A403 to residue D413, the “intrinsically disordered fragment”—IDF), which had not been determined in structure of human SK2/calmodulin complex (PDB: 5V02), was obtained in the predicted model (Figure 1A). It suggests that the predicted structure can be used to supplement the crystallized structure.

To test the accuracy of the predicted model, we used TM-align server to calculate the TM-align score (Zhang and Skolnick, 2005). The predicted models were aligned with 5V02, and the TM-align scores of SK2 and calmodulin are 0.99124 and 0.89215. These TM-align scores show that the structure of human SK2/calmodulin complex has been accurately predicted.

### 3.2. Binding Site Prediction

To determine the binding pocket in SK2/calmodulin complex, the Sitemap and LISE were exploited to predict binding sites (Halgren, 2007, 2009; Xie and Hwang, 2012). The top three predicted results from LISE were overlapped with the results of Sitemap. After the comparison, we found that there was only one consensus. Then we selected this binding site to generate receptor grid for docking. To verify the accuracy of binding site prediction, we also overlapped the predicted binding site with that of 5V02 (Figure 1B). Notably, the predicted binding site is the same binding site of Riluzole in 5V02, which suggests that this binding site is the potential binding site for the ligands. Hence, the *in silico* approaches successfully predict the accurate binding site.

### 3.3. Molecular Docking

**Figure 2:**
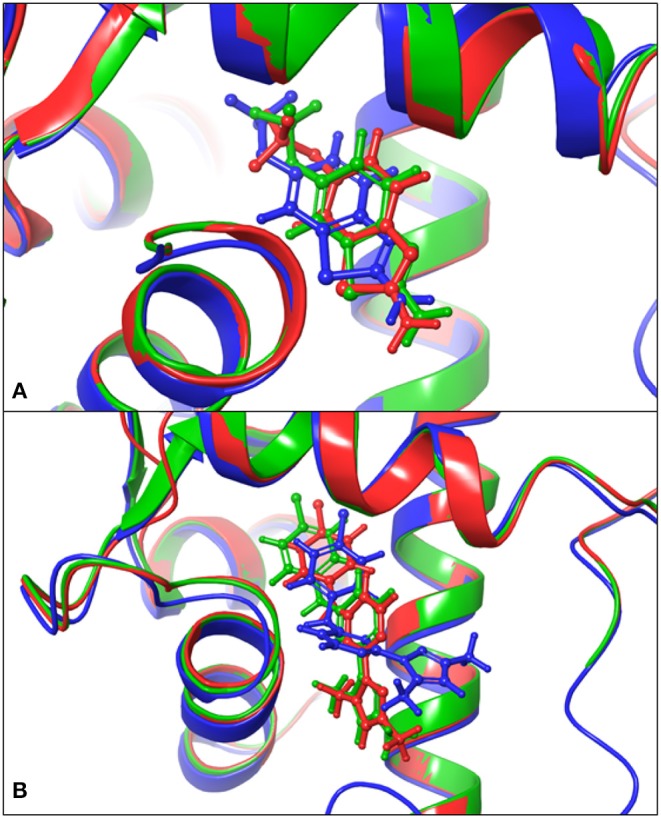
Comparison of the docked poses and the native structures. **(A)** Comparison of the docking results on the PSK2 in blue color, the docking results on 5V02 in green color, and determined structures 5V02 in red color; The blue-colored Riluzole is the docked pose on the PSK2, the green-colored Riluzole is the docked pose on 5V02, and the red-colored Riluzole is the determined structure in 5V02; **(B)** Comparison of the docking results on the PSK2 in blue color, the docking results on 5V03 in green color, and determined structure 5V03 in red color; The blue-colored CyPPA analog 1 is the docked pose on the PSK2, the green-colored CyPPA analog 1 is the docked pose on 5V03 and the red-colored CyPPA analog 1 is the determined structure in 5v03.

**Figure 3:**
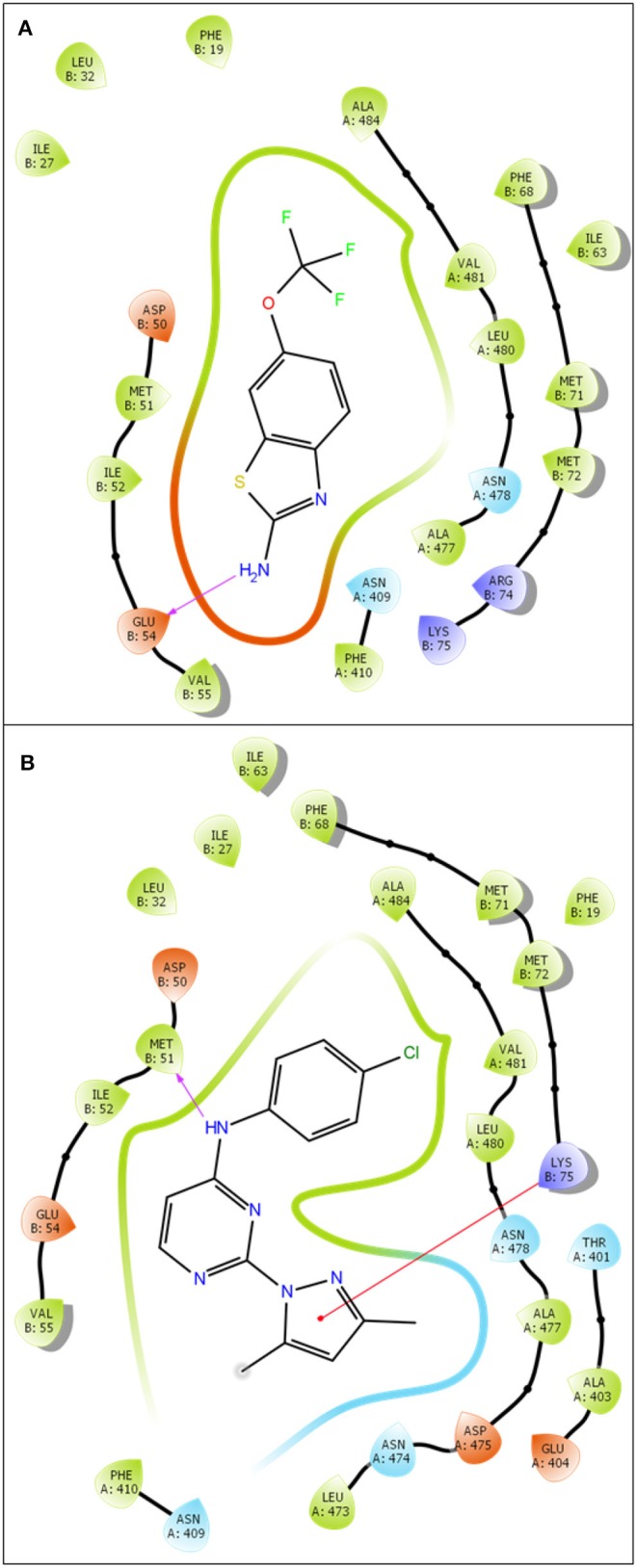
2D ligand-protein interaction of Riluzole **(A)** and CyPPA analog 1 **(B)** at PSK2 binding site. The pink arrow is referred to hydrogen bond. The red line is referred to Pi-cation interaction.

**Figure 4:**
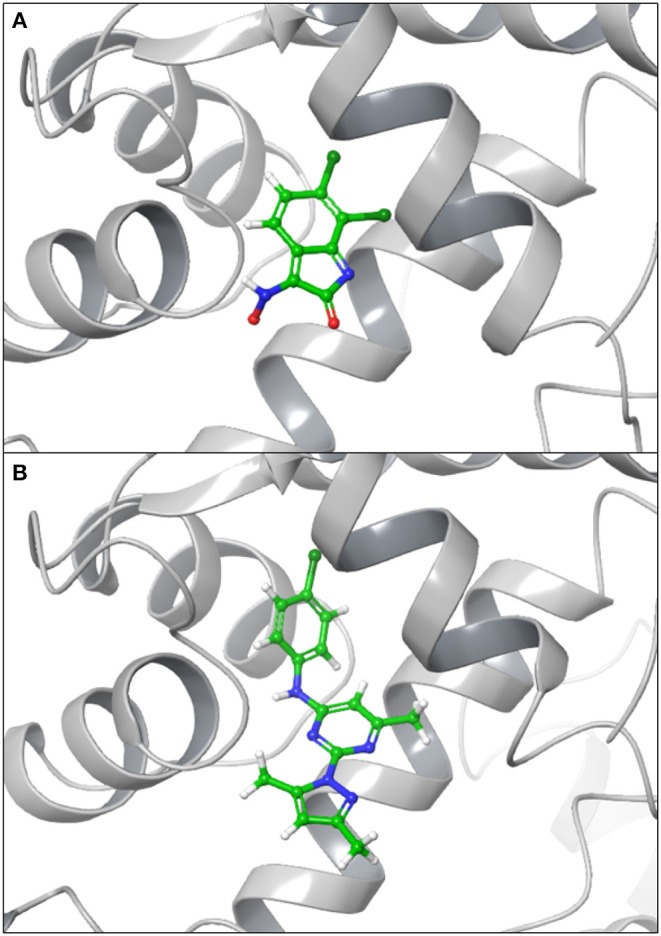
Docking poses of SK2 predicted structure with NS309 **(A)** and CyPPA analog 2 **(B)**.

Based on the previous study (Cho et al., 2018), the interface of SK2 and calmodulin can be bound by Riluzole, NS309, CyPPA analog 1, and analog 2. To investigate whether we can obtain the same results using *in silico* approaches, we first docked Riluzole and CyPPA analog 1 onto the predicted model (PSK2) via IFD (Induced Fit Docking) in Maestro, and redocked these ligands onto the determined structures (5V02 and 5V03) and calculated the binding energies as the control. Then we calculated the binding energies using MM-GBSA to estimate the binding affinities (Greenidge et al., 2012). As a result, the MM-GBSA ΔG_Bind_ of PSK2 with Riluzole and CyPPA analog 1 are −40.19 and −56.11 kcal/mol, respectively. As indicated in Table 1, those results of PSK2 are compatible to the results of 5V02 and 5V03, which demonstrate that accurate results can be obtained using *in silico* approaches. Additionally, the docking pose of PSK2 with Riluzole is almost identical with that in 5V02 (Figure 2A, the RMSD between the ligand of crystal structure and docking poses on 5V02 or PSK2 is 0.43 or 0.72 Å), which indicates that the simulated result from IFD can obtain accurate data in comparison with the results of crystallization. In Figure 2B, the coordinates of docked and native ligands are almost the same even though the poses of two ligands are not completely overlapped (the RMSD between the ligand of crystal structure and docking poses on 5V03 or PSK2 is 1.59 or 0.87 Å). Analyzing the docking results, Riluzole and CyPPA analog 1 interact with residues in both SK2 and calmodulin (Figure 3). The interacting residues in the binding site are mostly hydrophobic residues. As CyPPA analogs are larger molecules, they interact with more residues. Compared the list of interacting residues (Table S1), our results are almost identical to those of the previous study (Cho et al., 2018). The discrepancy may be because Maestro analyzes the interactions between ligands and the protein based on the interaction energy and the previous study (Cho et al., 2018) simply lists the residues within 5 Å of either ligand. According to the docking results of PSK2 with Riluzole and CyPPA analog 1, we docked NS309 and CyPPA analog 2 on the same binding site using the same methods mentioned above (Figure 4 and Figure S1). The binding affinities MM-GBSA ΔG_Bind_ are showed in Table 1. The MM-GBSA ΔG_Bind_ values of CyPPA analog 1 and CyPPA analog 2 on the PSK2 are more negative than those of the other ligands, which suggests that CyPPA analog 1 and CyPPA analog 2 are promising drug candidates.

**Table 1:** MM-GBSA ΔG_Bind_ of ligands bound to crystallized structures and predicted structures.

**Protein**	**Ligand**	**MM-GBSA ΔG_Bind_ (kcal/mol)**
5V02	Riluzole	−40.83
5V03	CyPPA analog 1	−62.72
PSK2	Riluzole	−40.19
PSK2	NS309	−49.97
PSK2	CyPPA analog 1	−56.11
PSK2	CyPPA analog 2	−64.84

**Figure 5:**
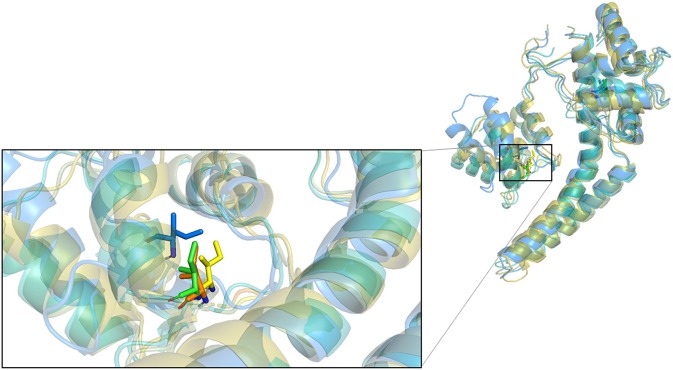
Superposition of four conformations of I100 at four time points on the simulation trajectory of the complex of PSK2 and NS309.

**Figure 6:**
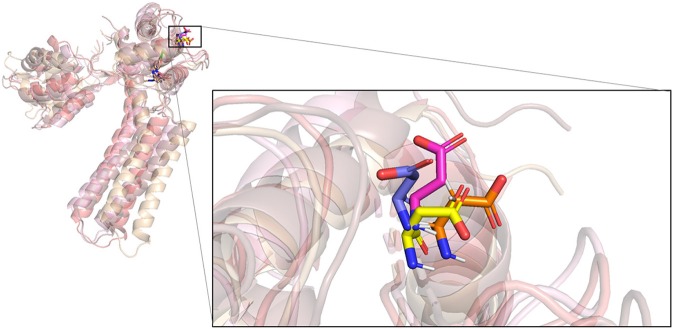
Superposition of four conformations of D64 at four time points on the simulation trajectory of the complex of PSK2 and CyPPA analog 1.

To verify the ligand-induced perturbations, NMR spectrum was used to identify residues with significant chemical shifts in previous experiments (Cho et al., 2018). With computational approaches, we ran MD simulations for each protein-ligand complex to simulate the conformational changes after the binding of ligands. A previous research reports that the residues on EF hands of calmodulin had conformational changes due to the ligand binding (Cho et al., 2018). Therefore, we calculated RMSD for those residues. According to the results of RMSD, we compared four trajectories of each residue and superposed their different poses at different time points. As a result, we found I100, on the complex of NS309, had obvious perturbations on four time points (Figure 5 and Figure S2). In addition, D64 which was a critical residue for calcium sensing, also showed dramatically conformational changing on the complex structure of PSK2 and CyPPA analog 1 (Figure 6 and Figure S3). Hence, the different poses of those residues demonstrated that the ligand could induce the perturbations of the calmodulin, which was consistent with the conclusion in the previous research (Cho et al., 2018).

### 3.4. Virtual Mutations at V481 and A477 in the Binding Pocket

The results of a previous study (Cho et al., 2018) show that the site-directed mutations on the key residues V481 and A477 in the binding site result in changes in the binding affinities. To validate that the *in silico* approach can simulate and predict the impacts of these mutations, we performed the virtual mutation experiments, docked the ligands on all mutants, and calculated the corresponding binding energies of each mutant. The MM-GBSA ΔG_Bind_ of V481 mutants are shown in Table 2. To verify the accuracy of *in silico* approaches, the Pearson's correlation coefficients between MM-GBSA ΔG_Bind_ and EC_50_ were calculated (*R* = 0.6 for NS309 in V481 mutants). Table 2 indicates that substitutions with small side chains (V481S) or a charged amino acid (V481D) significantly decrease the binding affinities of NS309. Conversely, the binding affinity of NS309 in PSK2 V481F is close to that in wild-type. The results above are consistent with the conclusion in literature, that is, this position requires a non-charged residue with a bigger side chain (Cho et al., 2018).

**Table 2:** EC_50_ and MM-GBSA ΔG_Bind_ of NS309 and CyPPA analog 2 bound to the V481 mutants.

**Protein**	**NS309**		**CyPPA analog 2**	
	**EC_50_ (μM)**	**MM-GBSA ΔG_Bind_ (kcal/mol)**	**EC_50_ (μM)**	**MM-GBSA ΔG_Bind_ (kcal/mol)**
WT	1.1	−49.97	2.5	−64.84
V481A	4.3	−54.28	>250	−29.05
V481S	5.1	−32.65	>250	−49.94
V481T	1.2	−32.77	46.6	−49.97
V481D	7.4	−25.97	>250	−46.75
V481F	0.8	−57.77	3.3	−64.09

Similarly, we find that CyPPA analog 2 in PSK2 V481F mutant with large aromatic side chains also shows a close binding affinity compared to that in PSK2 WT (Table 2), which demonstrates a good correlation between calculated binding affinities and EC_50_. The mutants with small side chains (V481A and V481S) or a charged amino acid (V481D) also shows the relatively lower binding affinities of CyPPA analog 2. Those results demonstrate that the simulation results are compatible with the data from biological experiments. The consistent conclusion has successfully proved that the all-computational protocol can be widely applied in future biomedical research.

In Table 3, all PSK2 A477 mutants have slightly lower NS309 potency than that of PSK2 WT and the predicted binding affinities MM-GBSA ΔG_Bind_ have similar results. According to the results in literature (Cho et al., 2018), CyPPA analog 2 should exhibit shifted potencies at PSK2 A477L, PSK2 A477V, PSK2 A477S, PSK2 A477T, PSK2 A477R, and PSK2 A477D, but not at PSK2 A477I. However, in Table 3, the MM-GBSA ΔG_Bind_ of PSK2 A477I is not different from those of other mutants. As an isoleucine has a long side chain, different rotamers may largely change the estimated binding affinities. Performing an MD simulation may be a good solution to optimize the structures of mutants and improve the docked poses and estimated binding energies.

**Table 3:** EC_50_ and MM-GBSA ΔG_Bind_ of NS309 and CyPPA analog 2 bound to the A477 mutants.

**Protein**	**NS309**		**CyPPA analog 2**	
	**EC_50_ (μM)**	**MM-GBSA ΔG_Bind_ (kcal/mol)**	**EC_50_ (μM)**	**MM-GBSA ΔG_Bind_ (kcal/mol)**
WT	1.1	−49.97	2.5	−67.14
A477I	2.0	−37.26	2.0	−47.89
A477L	1.7	−24.71	99.0	−37.60
A477V	2.0	−39.67	>250	−45.63
A477S	3.0	−36.65	>250	−46.05
A477T	2.0	−23.36	>250	−51.04
A477R	2.1	−31.24	>250	−51.50
A477D	3.9	−30.94	>250	−49.71

## 4. Discussion

In the field of new drug discovery, research efficiency is particularly essential. On one hand, the speed of new drug development is of great importance to patients, especially the ones with fatal diseases such as cancers or acute infectious diseases (Shi et al., 2015). Statistics show that there will be around 1.7 million new cancer cases and 600 thousands cancer deaths in the United States in 2019 (Siegel et al., 2019). This race against time has always been a huge challenge for the researchers in this field. On the other hand, to bring a new drug to the market from compound identification to final FDA approval usually costs up to billions of dollars (Cleary et al., 2018). Therefore, the time and cost-efficient virtual process of drug development will benefit many pharmaceutical companies and our society. *In silico* approaches which can save considerable amount of research time and cost should be applied to drug design. With the rapid development of computer science and engineering, the availability and accuracy of *in silico* approaches have been constantly improving. Although many progresses have been made in utilizing *in silico* approaches to simulate certain biological experiments, the whole experimental processes completed in the virtual way from protein structure simulation to protein-drug interaction characterization has never been achieved before.

In this study, we proposed a protocol of integration of *in silico* approaches to simulate the process from protein structure determination to key residues mutagenesis and characterization. To validate this strategy, we selected the human SK2 ion channels as our target protein. With successful prediction, we obtained accurate protein structures (TM-align score >0.5) and the same binding site as the crystallized structure. Furthermore, the docking poses of Riluzole and CyPPA analog 1 are consistent with the ligand bound conformations in the crystallized structures. We also successfully reproduced similar effects of site-directed mutagenesis on the ligand binding, which demonstrated great potential of the integration of *in silico* approaches. However, the purpose of integrating *in silico* approaches is not to completely replace biological experiments but to speed up the drug discovery process with the continuous and automatic computational process.

A possible reason why an all-computation protocol of drug design has not been proposed and implemented is the inaccuracy or uncertainty of prediction results might accumulate in the sequential computational pipeline. However, the state-of-the-art bioinformatics algorithms, software or servers have been highly accurate in many cases so that it is time to integrate them into a fully computational process or even a fully automatic process. This is the first study to demonstrate the feasibility of an all-computational protocol in drug design. To achieve the ultimate goal of “automatic” drug discovery, more online servers or computational algorithms like PROCHECK (Laskowski et al., 1993), which can be used to assess or estimate the reliability of prediction results generated by each *in silico* approach, need to be developed. Despite its innovative approach, there are a few limitations of this study. First, the accuracy of protein structure prediction relies on the methods and/or templates. In our research, we selected the SK2/calmodulin from *Rattus norvegicus* (PDB ID: 4J9Z) as the template to build protein structure on SWISS MODEL, whose results are more accurate than the results from other webservers (data not shown). Hence, a reliable tool or method is critical to the accuracy of the final simulated results. Second, the binding affinity changes are hard to be precisely reproduced, especially those of the mutants, because considering possible conformational changes on target proteins is still the biggest challenge of docking. This suggests that MD simulation which can simulate the conformational changes should be used to further improve the precision of the predictions.

In conclusion, this work established and demonstrated an integrated protocol of *in silico* approaches for the first time. Its applicability can be potentially extended beyond the characterizing of SK2 ion channels to investigating other proteins with unknown structures, such as the Alzheimer's disease related proteins (Fitzpatrick et al., 2017; Hatami et al., 2017), which are also treatment targets of neural degenerative diseases. Although there are challenges to the *in silico* approaches, our work still paves a new way toward an automatic procedure of drug design.

## Data Availability Statement

All datasets generated for this study are included in the article/Supplementary Material.

## Author Contributions

YW carried out the research, analyzed the data, and drafted the manuscript. LL analyzed the data. Z-RX conceived the project, guided the research, and revised the manuscript. All authors read and approved the final manuscript.

### Conflict of Interest

The authors declare that the research was conducted in the absence of any commercial or financial relationships that could be construed as a potential conflict of interest.
