# Supplementary material for: A Pilot Study of All-Computational Drug Design Protocol–From Structure Prediction to Interaction Analysis
Source: Front Chem. 2020 Feb 12;8:81. doi: 10.3389/fchem.2020.00081 (PMC7028743; doi:10.3389/fchem.2020.00081)
Supplement: Supplementary file 1 [file Table_1.DOCX]

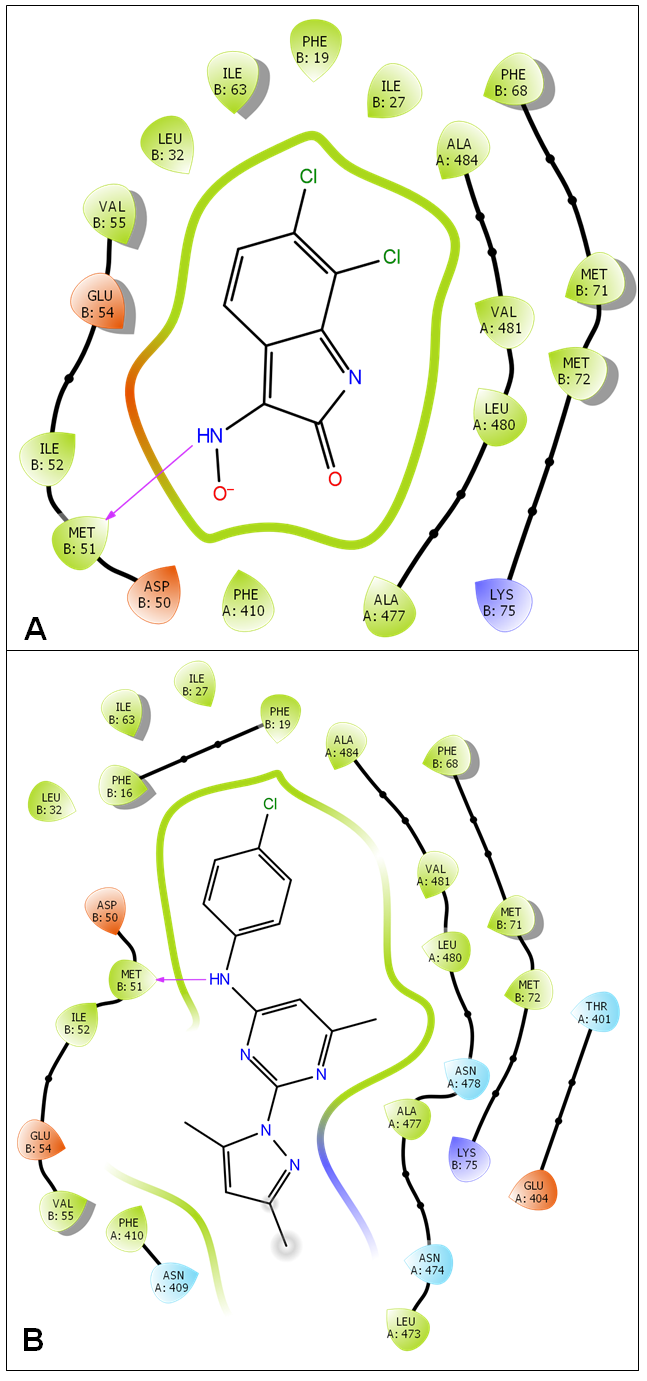


**Figure S1. 2D ligand-protein interaction of NS309 (A) and CyPPA analog 2 (B) at PSK2 binding site**. The pink arrow is referred to hydrogen bond.


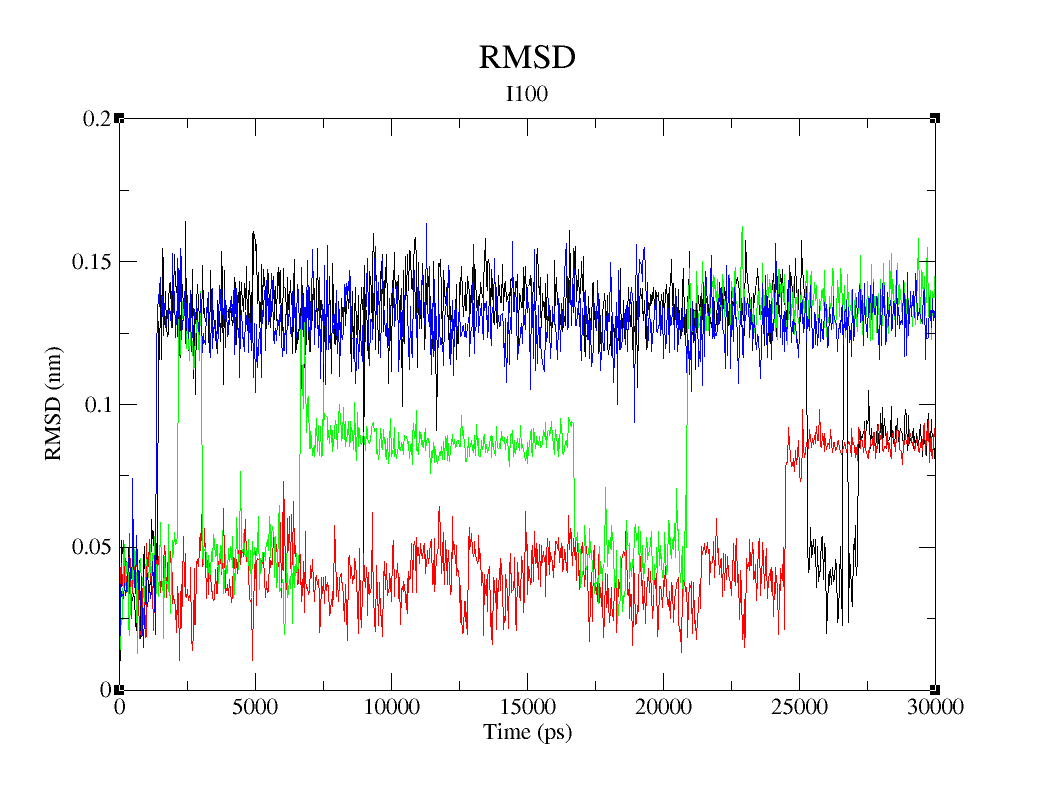


**Figure S2. Root mean square deviation (RMSD) of I100 in PSK2 apo structure and its three complexes during the 30 ns molecular dynamics simulation.** The black line indicates the apo structure; the red line indicates Riluzole bound complex; the green line indicates NS309 bound complex; the blue line indicates CyPPA analog 1 bound complex. The trajectories of apo structure and NS309 binding complex (black and green lines) are significantly different. We chose the conformations at four time points based on these trajectories.


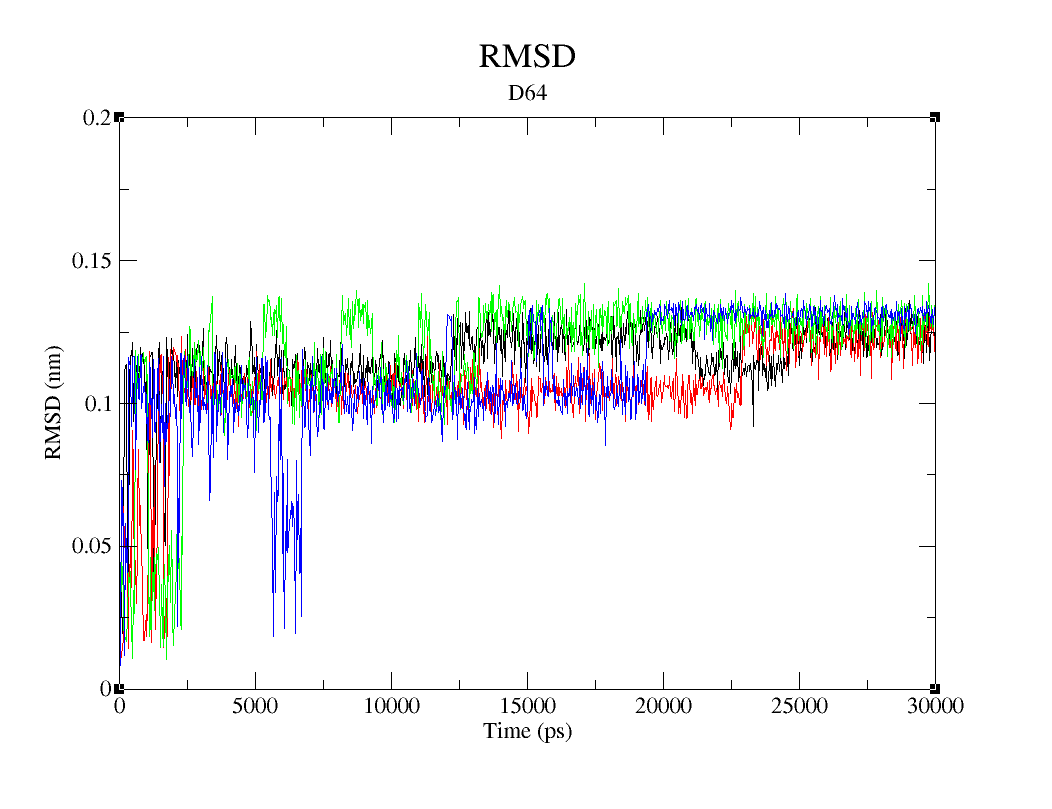


**Figure S3. Root mean square deviation (RMSD) of D64 in PSK2 apo structure and its three complexes during the 30 ns molecular dynamics simulation.** The black line indicates the apo structure; the red line indicates Riluzole bound complex; the green line indicates NS309 bound complex; the blue line indicates CyPPA analog 1 bound complex. The trajectories of apo structure and CyPPA analog 1 binding complex (black and blue lines) are significantly different. We chose the conformations at four time points based on these trajectories.

Table S1. The Binding Surfaces of CyPPA analog 1 and Riluzole in crystallized structures and predicted structures.

| **CyPPA analog 1 Binding Surface (<5Å SK2, 5V03)** | **Riluzole Binding Surface (<5Å SK2, 5V02)** | **CyPPA analog 1 Binding Surface (<5Å PSK2)** | **Riluzole Binding Surface (<5Å PSK2)** |
| --- | --- | --- | --- |
|  |  | T401 |  |
|  |  | A403 |  |
|  |  | E404 |  |
|  | N409 (4.8) | N409 | N409 |
|  | F410 (3.7) | F410 | F410 |
| Q470 (3.6) |  |  |  |
| L473 (4.4) |  | L473 |  |
| N474 (3.7) |  | N474 |  |
|  |  | D475 |  |
| A477 (3.5) | A477 (3.9) | A477 | A477 |
|  |  | N478 | N478 |
| L480 (3.6) | L480 (3.3) | L480 | L480 |
| V481 (4.5) | V481 (3.5) | V481 | V481 |
|  |  | A484 | A484 |
| **CyPPA analog 1 Binding Surface (<5Å Calmodulin-5V03)** | **Riluzole Binding Surface (<5Å Calmodulin-5V02)** | **CyPPA analog 1 Binding Surface (<5Å Calmodulin-PSK2)** | **Riluzole Binding Surface (<5Å Calmodulin-PSK2)** |
| F19 (3.7) | F19 (3.9) | F19 | F19 |
| I27 (4.4) | I27 (4.2) | I27 | I27 |
| L32 (4.0) | L32 (3.8) | L32 | L32 |
| D50 (3.9) |  | D50 | D50 |
| M51 (3.1) | M51 (3.6) | M51 | M51 |
| I52 (4.6) |  | I52 | I52 |
| E54 (3.2) | E54 (2.3) | E54 | E54 |
| V55 (3.5) | V55 (4.6) | V55 | V55 |
| I63 (3.5) | I63 (3.2) | I63 | I63 |
| F68 (3.5) | F68 (3.5) | F68 | F68 |
| M71 (3.8) | M71 (2.5) | M71 | M71 |
|  |  | M72 | M72 |
|  |  |  | R74 |
|  |  | K75 | K75 |
